# Nurses’ Experiences After Implementation of an Organization-Wide Electronic Medical Record: Qualitative Descriptive Study

**DOI:** 10.2196/39596

**Published:** 2022-07-26

**Authors:** Rebecca M Jedwab, Elizabeth Manias, Alison M Hutchinson, Naomi Dobroff, Bernice Redley

**Affiliations:** 1 Centre for Quality and Patient Safety Research-Monash Health Partnership Institute for Health Transformation School of Nursing and Midwifery, Deakin University Melbourne Australia; 2 Nursing and Midwifery Informatics Monash Health Melbourne Australia; 3 Institute for Health Transformation School of Nursing and Midwifery, Deakin University Melbourne Australia; 4 Nursing and Midwifery, Monash Health Melbourne Australia; 5 School of Nursing and Midwifery, Deakin University Melbourne Australia

**Keywords:** computerized medical records systems, COVID-19, implementation science, motivation, nurses, qualitative research

## Abstract

**Background:**

Reports on the impact of electronic medical record (EMR) systems on clinicians are mixed. Currently, nurses’ experiences of adopting a large-scale, multisite EMR system have not been investigated. Nurses are the largest health care workforce; therefore, the impact of EMR implementation must be investigated and understood to ensure that patient care quality, changes to nurses’ work, and nurses themselves are not negatively impacted.

**Objective:**

This study aims to explore Australian nurses’ postimplementation experiences of an organization-wide EMR system.

**Methods:**

This qualitative descriptive study used focus group and individual interviews and an open-ended survey question to collect data between 12 and 18 months after the implementation of an EMR across 6 hospital sites of a large health care organization in Victoria, Australia. Data were collected between November 2020 and June 2021, coinciding with the COVID-19 pandemic. Analysis comprised complementary inductive and deductive approaches. Specifically, reflexive thematic analysis was followed by framework analysis by the coding of data as barriers or facilitators to nurses’ use of the EMR using the Theoretical Domains Framework.

**Results:**

A total of 158 nurses participated in this study. The EMR implementation dramatically changed nurses’ work and how they viewed their profession, and nurses were still adapting to the EMR implementation 18 months after implementation. Reflexive thematic analysis led to the development of 2 themes: *An unintentional divide* captured nurses’ feelings of division related to how using the EMR affected nurses, patient care, and the broader nursing profession. *This time, it’s personal* detailed nurses’ beliefs about the EMR implementation leading to bigger changes to nurses as individuals and nursing as a profession than other changes that nurses have experienced within the health care organization. The most frequent barriers to EMR use by nurses were related to the Theoretical Domains Framework domain of environmental context and resources. Facilitators of EMR use were most often related to memory, attention, and decision processes. Most barriers and facilitators were related to motivation.

**Conclusions:**

Nurses perceived EMR implementation to have a mixed impact on the provision of quality patient care and on their colleagues. Implementing technology in a health care setting was perceived as a complex endeavor that impacted nurses’ perceptions of their autonomy, ways of working, and professional roles. Potential negative consequences were related to nursing workforce retention and patient care delivery. Motivation was the main behavioral driver for nurses’ adoption of EMR systems and hence a key consideration for implementing interventions or organizational changes directed at nurses.

## Introduction

### Background

The implementation of health care technology systems such as electronic medical record (EMR) systems causes major changes in nurses’ workplaces, work, and workflows [1]. Understanding EMR-related impact on nurses is needed to support their work in providing round-the-clock, direct patient care [2]. The implementation of new health care technologies has been shown to increase nurses’ stress [3-5]; hence, such implementation may exacerbate existing problems of nurse shortages and high workloads. There is an urgent need to understand ways to support nurse retention and productivity in rapidly changing workplaces [6]. In contrast to international settings, the implementation of EMR systems throughout Australian hospitals has been relatively recent and has been promoted as enabling safe patient care [7] rather than financial incentivization. Examination of the impact of EMR implementation on nurses is limited and has largely focused on aspects of EMR implementation such as integrating EMR knowledge and exposure into university nursing education [8] or EMR usability [9]. Although EMR usability is often an enabler of EMR use and uptake, the study by Lloyd et al [9] focused on the comparison between nurses and medical colleagues and differences between clinical settings, rather than nurses’ experiences. Nurses’ attitudes toward health care technology were the focus of an integrative review of Australian literature; however, the scope of the review by Mills et al [10] was broader than EMR systems and identified mixed outcomes related to nurses’ attitudes toward health care technologies (both positive and negative perceptions of usefulness and impact on patient care) [10]. Although 1 observational time and motion study examined an Australian health care organization’s large-scale EMR implementation, the focus was on time spent on nursing activities and time spent with patients and not on the nurses’ experiences of using the system [11]. Another Australian qualitative study explored nurses’ early experiences after EMR implementation [12]; however, the EMR was a hybrid system (mix of paper and electronic documentation) and therefore a gap remains in understanding nurses’ experiences of a large-scale full EMR implementation in their workplace [13]. There is a need to investigate nurses’ experiences of an EMR implementation to ensure any changes to nurses’ work do not impact the delivery of safe, high-quality patient care. In the qualitative study underpinning this paper, Australian nurses’ postimplementation experiences of an organization-wide complete EMR system were examined inductively and deductively, using a theoretical framework to support the understanding and contextualization of its influences on nurses’ behaviors.

### Purpose

As EMRs become commonplace throughout Australian health care organizations and demands on nurses increase, it is vital to explore nurses’ experiences and monitor and mitigate negative impacts on nurses’ work and workflows and the nurses themselves. This study aimed to explore Australian nurses’ EMR experiences after implementation of an organization-wide EMR system to inform future technology implementation strategies that enhance nurses’ work, workflows, and well-being.

## Methods

### Overview

This qualitative descriptive study used data collected from focus group and individual interviews and free-text responses to an open-ended question at the end of a survey: “Please use the box below for any additional comments on your experiences of EMR.” Qualitative data were collected in the context of a large mixed methods pre- and postimplementation study. Preimplementation qualitative data and pre-post survey findings have been reported elsewhere [14-16].

### Inclusion and Exclusion Criteria

All nurses working in inpatient areas throughout 6 hospital sites of a single health care organization where the EMR was implemented in 2019 were eligible for inclusion. Nurses working casually (ie, not in a permanent position) as part of the EMR implementation team or working in areas where the EMR system was not fully implemented (ie, using a combination of paper and electronic documentation) were excluded.

### Setting, Recruitment, and Data Collection

A large multisite public health care organization located in Victoria, Australia, was the setting for this study. The health care organization provides public health care services to persons of all ages in inpatient and outpatient settings, employs over 8500 nurses and midwives, and has approximately 3300 beds across major hospital sites and multiple community locations. The service caters to a multicultural, linguistically and sociodemographically diverse community. The Australian health care system provides public health insurance coverage for all, with the option for individuals to purchase private health insurance if desired [17]. The health care organization implemented the EMR system in 3 stages during 2019: one site went live in August 2019, two sites in October 2019, and three sites in November 2019.

Recruitment for participation in the post-EMR implementation phase included several strategies: nurses provided their email address at the end of a survey indicating their consent to be contacted to participate in qualitative data collection; nurses were invited to provide free-text comments at the end of the survey; and real-time in-person convenience sampling recruitment was undertaken in clinical settings. Using these multiple recruitment strategies helped to minimize the intrinsic limitations of convenience sampling, limited response bias, and supported broad participation in data collection. All nurses who met the eligibility criteria and indicated interest, verbally or in writing, were given an opportunity to participate.

Data collection occurred between November 2020 and June 2021. Throughout this period, access to the hospital sites varied owing to restrictions associated with the COVID-19 pandemic. Web-based platforms were used when in-person access was restricted. In-person data collection complied with COVID-19 pandemic–related health care and social distancing restrictions. All focus group and individual interviews were conducted by the first author (RMJ; female PhD student employed at the health care organization) at mutually agreeable times that included after hours and weekends. To acknowledge their time for participation, a drink voucher was offered to nurses at the completion of the focus group or individual interviews (approved by ethics committees). None of the nurses withdrew consent or participated more than once.

The semistructured interview guide was based on the “4I” model for appreciative inquiry [18] and included open-ended questions such as “How are you feeling about the EMR?” and “What works well?” The same interview guide was used for all focus group and individual interviews (Multimedia Appendix 1). Nurses had the option of providing demographic information, including their age, gender, nurse classification, years of work experience, highest education level, hours worked per fortnight, clinical work area, and the specific site of the health care organization. In addition, the free-text survey comments provided in response to the statement “Please use the box below for any additional comments on your experiences of EMR” were included in the analysis. The researcher (RMJ) collected field notes and reflective notes to ensure reflexivity. All interviews were recorded and transcribed verbatim for analysis. Nurse participants were not invited to review transcripts or results owing to logistic issues of repeated contact and privacy concerns and to minimize any burden.

### Study Rigor

Study trustworthiness incorporated consideration of study credibility, dependability, transferability, and reflexivity [19]. Credibility was upheld by including multiple research team members in data analysis. The use of illustrative quotes, research team involvement in discussions of data interpretation and analysis, and keeping detailed field notes supported study and data dependability. Transferability was supported by broad inclusion criteria and recruitment of diverse nurse participants across multiple hospital sites. Research team reflexivity was discussed and managed through reflection on roles, biases, and perceptions in relation to data interpretation and analysis [20].

### Theoretical Framework

Justification for using the Theoretical Domains Framework (TDF) [21] and Capability, Opportunity, Motivation-Behavior (COM-B) model [22] as an analytical framework and model for deductive analysis in this study is 4-fold: First, there is a lack of theory-informed investigations examining Australian nurses’ experiences of EMR adoption in their workplace. Second, the use of this theoretical framework and model to examine barriers to and facilitators of nurses’ EMR use supports the identification of targeted theory-informed behavior change interventions that address specific barriers and promote desired behaviors [23]. Third, the analytical framework and model have been widely and successfully used to evaluate and design interventions related to health care settings and digital health [24,25]. Finally, the TDF considers a wide range of individual, organizational, and contextual factors that affect behavior; hence, it assists in exploring and understanding behavioral influences on nurses’ postimplementation EMR experiences.

### Data Analysis

#### Overview

Complementary inductive and deductive data analysis methods over 2 stages were used to create meaning from the data and elicit a deep understanding of nurses’ perspectives and experiences post-EMR implementation. All qualitative data were included in both stages of inductive and deductive data analysis. Inductive data analysis using reflective thematic analysis [20] was completed first to minimize the risk of fitting data to a predetermined idea or model [26] and to explore and capture meaning across the data set [27]. In the second analytical stage, the TDF was used as a deductive theoretical coding framework to support the understanding of factors that affected nurses’ behaviors in the post-EMR implementation phase [21].

#### Inductive Data Analysis

The following six steps of reflective thematic analysis by Braun and Clarke [20] were used to guide the inductive data analysis:

1. Familiarization: data familiarization included listening to the audio recordings multiple times and multiple readings of the interview transcripts and qualitative comments. Field comments and reflexive notes were also reviewed.
2. Coding: data were transferred into Microsoft Excel (version 2019), and each quote was defined as up to 3 sentences long. Coding was undertaken inductively by the first author in 2 rounds and then grouped into subthemes and themes.
3. Generating initial themes.
4. Developing and reviewing themes.
5. Refining, defining, and naming themes: regular discussions with the research team throughout data collection and data analysis were used to support coding, subtheme and theme development, refinement and naming, and discussion of data saturation. The lack of new information was reached by the end of the 19th interview; however, data collection continued to ensure that opportunities were given for all nurses interested in participating.
6. Writing-up: the findings of thematic analysis are included in their entirety in this paper.

#### Deductive Data Analysis

After the completion of inductive data analysis, deductive data analysis commenced with the coding of each quote to one of the 14 domains of the TDF [21]. Context was used to identify each code as either a barrier to or facilitator to nurses’ use of the EMR. The TDF data were subsequently mapped to the corresponding COM-B components [22].

### Ethics Approval and Data Reporting

Approval (low-risk) was obtained from both Monash Health and Deakin University Human Research Ethics Committees (references HREC/46439/MonH-2018-154603(v3) and 2019-003). The Consolidated Criteria for Reporting Qualitative Studies guidelines were used to guide data reporting [28].

## Results

### Overview

A total of 158 nurses participated in this study: 35 (22%) nurses participated through focus group interviews (6/22, 27%) or individual interviews (16/22, 73%). The survey, sent to 4159 nurses, had a response rate of 9.5%. A total of 31.4% (123/392) of survey respondents provided free-text responses to an open-ended question that were analyzed in conjunction with focus group and individual interview data. In total, 20% (78/392) of survey respondents provided their email addresses, but despite initial contact and 2 reminder emails, only 22% (17/78) of these nurses participated in a focus group or individual interview. A total of 36% (8/22) of interviews were conducted on-site at the health care organization and the remainder were web-based (14/22, 64%). The focus group or individual interviews lasted between 12 and 70 (median 32) minutes, with up to 9 nurses per focus group. Nurses who did not wish to provide their demographic information or refused audio recording but wished to participate through the collection of field notes were permitted to do so.

### Participant Demographics

Participant demographics information was available for 146 nurses. Most were registered nurses (45/146, 30.8%), aged between 50 and 59 years (40/146, 27.4%), female (132/146, 90.4%), had worked between 4.5 and 9 years (24/146, 16.4%), had a postgraduate qualification (55/146, 37.7%), and worked part-time (60/146, 41.1%) in critical care areas (74/146, 50.7%) of the health care organization (Multimedia Appendix 2).

### Nurses Adopting and Adapting to the EMR

The first stage of data analysis involved inductive data analysis using reflexive thematic analysis and led to the development of two overarching themes: (1) *An unintentional divide*; and (2) *This time, it’s personal*. Exemplar quotes for each theme and subtheme are presented in Multimedia Appendix 3.

#### Theme 1: An Unintentional Divide

The implementation of the EMR system caused feelings of division among nursing staff related to the implementation and adoption of EMR; ongoing support; perceptions of EMR and how it affected their work; and how EMR impacted the nurse as an individual and their profession. *An unintentional divide* includes three subthemes: (1) Then and now, (2) Clicking or caring, and (3) Consequences and assumptions.

##### Subtheme 1: Then and Now

*Subtheme 1: Then and Now* contrasts nurses’ reflections on their initial reactions to EMR implementation processes and adapting to the new system. Nurses reflected on missed opportunities to improve clinical practice with EMR compared with paper-based systems and discussed different social influences that impacted their experience of adapting to a new way of working. Nurses also discussed the differences in how they thought the EMR was implemented, that is, whether they thought it was beneficial to change all clinical documentation over to a computer system at once, and factors that influenced their experiences and helped them to adapt over time, such as culture, training, and support (offered both during implementation and on an ongoing basis).

Nurses referred to differences between their initial reactions to the implementation process and support provided at the time of implementation “then” and “now” when data collection occurred (between 12 and 18 months after implementation). Nurses felt that their initial reactions (shock, disappointment, and stress) had developed over time into some level of acceptance (learning about different aspects of EMR and getting used to working with computers).

Nurses discussed their unmet expectations related to specific aspects of the EMR implementation, such as eliminating documentation duplication and expanding nurses’ scope of practice. Some nurses felt their expectations were not met with the EMR and found the implementation period stressful; hence, it took them time to adjust (Multimedia Appendix 3, quotes 1-4). The timing of the EMR implementation, and if all clinical documentation were transitioned to the EMR at once, it affected whether some nurses deemed it as a positive or negative experience (Multimedia Appendix 3, quote 5).

Nurses also discussed the factors that influenced their experiences and adaptation to the new system, such as attitudes and culture, support, training, and education. Nurses felt that the implementation was both positively and negatively affected by the attitudes of their colleagues and the ward culture, as well as whether nurses were confident in using technology (Multimedia Appendix 3, quotes 6-8). Some nurses admitted that getting used to the system was difficult and took some time; however, as time went on, they adapted and learned. Nurses acknowledged that EMR adoption was more difficult for colleagues who worked part-time and therefore lacked EMR experience or exposure (Multimedia Appendix 3, quotes 9-11, 17, and 18).

The EMR implementation was described as successful by nurses when they felt that they had learned the system and adapted their ways of working and workflows. Many nurses attributed this success to the ongoing support provided by the organization throughout the EMR implementation and were grateful. Nurses also identified super users (nurses who had undergone increased EMR training and education and provided collegial support specific to EMR implementation) and their training as valuable and supportive (Multimedia Appendix 3, quotes 14 and 15). However, it should be noted that some super users acknowledged that they experienced difficulty and stress, which they attributed to pressure to support their peers with the EMR while also caring for their own patients (Multimedia Appendix 3, quote 16). At the time of data collection, some nurses thought that the EMR could be better used to its full capacity, citing knowledge gaps from inadequate training and ongoing education, and poor understanding of new EMR workflows impacted its use (Multimedia Appendix 3, quote 12). Nurses provided suggestions for how the health care organization could better support their workforce during an EMR implementation, as well as suggestions to improve EMR acceptance that often referred to individualization of the screen or alerts (Multimedia Appendix 3, quote 19).

##### Subtheme 2: Clicking or Caring

This subtheme reflected nurses’ different feelings about what they should spend their time on the EMR or their patients. With limited time, some nurses felt they faced a choice between having “to click” (ie, use the EMR) versus “care” (ie, spending time on patient care). Some nurses felt that their duty toward the EMR took them away from direct patient care and interpersonal interactions (Multimedia Appendix 3, quotes 20 and 21). Others expressed concerns that colleagues were completing EMR documentation that did not match their clinical actions because they were worried about the potential for negative responses from management (Multimedia Appendix 3, quotes 22 and 23).

Nurses often expressed both positive and negative views about EMR workflows and their impact on nurses (eg, device integration not being available for all clinical areas, communication changes, and medication safety concerns related to visibility and clarity of orders on EMR; Multimedia Appendix 3, quotes 24-30). Nurses reported negative feelings related to poor experiences with the privacy of patient information with the EMR (ie, anyone being able to read the computer screen), not finding the EMR easy to use, the layout of the EMR, and vast amounts of information (Multimedia Appendix 3, quotes 34-36). Negative experiences of using the EMR caused nurses to develop workarounds to circumnavigate EMR aspects they were unhappy with or that did not fit their desired way of working (Multimedia Appendix 3, quotes 37 and 38). This negativity was often voiced in the context of the time taken to perform nursing tasks or documentation, time spent finding out where to document on the EMR, or correcting documentation from other colleagues (Multimedia Appendix 3, quotes 39-42). In contrast, nurses’ positive EMR experiences related to patient care delivery included the ability to view all clinical information in one location, improved legibility owing to eliminating handwriting, and less duplication of documentation (Multimedia Appendix 3, quotes 32 and 33).

Nurses felt that their autonomy had been negatively impacted by EMR and that their clinical documentation on the EMR was of lower quality than when it was on paper (Multimedia Appendix 3, quotes 43 and 44). The EMR was perceived as both a physical and psychological barrier to providing patient care, and the EMR hardware and software disrupted nursing care (Multimedia Appendix 3, quotes 41, 42, and 45-48). When asked what matters most, nurses most often responded that nursing documentation quality and meaningfulness in the context of providing quality and safe patient care (Multimedia Appendix 3, quotes 49 and 50).

##### Subtheme 3: Consequences and Assumptions

Consequences and assumptions captured the impact of the EMR implementation on nurses’ work satisfaction and well-being, and how this affected different groups of nurses. Many nurses reported how they were impacted by the EMR implementation. Some attributed their decrease in work satisfaction since EMR implementation to having less time with patients and experiencing changes to their work and interpersonal relationships.

Negative changes in nurses’ work satisfaction and personal well-being also had negative impacts on their intention to remain in the workforce (Multimedia Appendix 3, quotes 51-53). Some nurses’ comments indicated that they were questioning whether they remained in their roles, whereas others stated that they knew of nurses who had resigned from the organization owing to the pressure associated with EMR implementation. There were nurses who acknowledged that there were other work stressors, not just EMR, contributing to nurses’ decreased well-being and work satisfaction, including the COVID-19 pandemic. The pandemic was often referred to as an additional burden on nurses and appeared to exacerbate the stress of EMR owing to requiring personal protective equipment to work in isolation rooms while using the EMR (Multimedia Appendix 3, quotes 54 and 55). Conversely, other nurses acknowledged that having the EMR was useful during the SARS-CoV-2 pandemic, providing up-to-date clinical information accessible to the entire health care team (Multimedia Appendix 3, quote 56).

Differences in nurses’ assumed or actual EMR knowledge created unrest among groups of nurses, including older and younger nurses, and those who were more knowledgeable or proficient with the EMR and those who were not. Nurses felt that some colleagues who were not competent or confident in using the EMR negatively impacted them because of subsequent challenges with documentation, where missing information from previous shifts made it difficult for nurses to complete their work. Some nurses reported that they felt divided from their colleagues, and they were being judged on their EMR ability, not their clinical knowledge or nursing experience (Multimedia Appendix 3, quotes 57-59). Judgment related to EMR knowledge and capability extended to assumptions about groups of nurses, specifically related to age. When referring to younger nurses, it was assumed they were more computer-literate and therefore would be more competent with EMR. Older nurses were acknowledged as valuable peers who were not as competent with technology as their younger colleagues and felt the largest negative impact of the EMR implementation (Multimedia Appendix 3, quotes 60-62). However, some nurses expressed surprise that there were older nurses coping with the EMR implementation (Multimedia Appendix 3, quote 63).

#### Theme 2: This Time, It’s Personal

Nurses reported that the EMR implementation was a bigger change and had had more personal consequences for both the nurse individually and nursing as a profession than other workplace changes they had experienced. *This time, it’s personal* includes two subthemes: (1) A constantly changing profession and (2) What will nursing become?

##### Subtheme 1: A Constantly Changing Profession

*Subtheme 1: A constantly changing profession* reflects nurses’ experiences of the change of an EMR and how the EMR implementation compared with previous workplace changes they had experienced. Nurses also discussed their fears and frustrations with the changes introduced by the EMR implementation.

Many nurses felt that as a profession, nurses were used to change; however, EMR implementation appeared to have a greater impact on their professional practice than previous changes they had experienced (Multimedia Appendix 3, quotes 64 and 65). Nurses discussed their colleagues’ different responses, their resistance to change, and how the change affected other nurses, leading to a loss of confidence. The physical change to electronic documentation on EMR and discontinuation of paper-based documentation appeared to be what mostly challenged nurses, although some nurses noted it was a positive change owing to the reduced incidence of lost information and poor communication (Multimedia Appendix 3, quotes 66-69). Some nurses felt that the changes increased their time spent on documentation, and this was often compared with other EMR systems perceived as easier to use (Multimedia Appendix 3, quotes 70 and 71).

Nurses acknowledged the inevitability of moving to an electronic documentation system, several fears and frustrations with the system and workflows upset nursing staff, including potential negative consequences for themselves individually and for nursing as a profession. Fear of negative consequences owing to changes to the visibility and legality of nursing documentation was often voiced, with nurses’ frustration extending to having to use and adapt to a system that they felt was not easy or intuitive to use (Multimedia Appendix 3, quotes 74-78). Unfortunately, despite extensive consultation with nurses across clinical areas throughout the health care organization, some felt there was insufficient nursing input in the development of the EMR. (Multimedia Appendix 3, quotes 79 and 80).

##### Subtheme 2: What Will Nursing Become?

*Subtheme 2: What will nursing become?* reflects nurses’ concerns regarding perceptions of their roles in this new era of technology use. Nurses expressed concern about the increasing busyness of nurses’ work and how the EMR would impact this busyness, and were also unsure how the EMR would change the perceptions and roles of their profession. However, nurses were also grateful to be able to provide their opinions regarding the EMR and to provide feedback and suggestions regarding EMR optimization.

Nurses were worried that their valuable story and insight, which came only from the unique role of providing 24/7 patient care, was missing from the EMR (Multimedia Appendix 3, quotes 81 and 82). To some nurses, their work, and the visibility of their work had become more task focused with the EMR, and they felt that nursing had become depersonalized. Nurses also expressed the view that some colleagues from other disciplines have different views of clinical documentation on EMR and felt this changed the relationships among professions (Multimedia Appendix 3, quotes 83 and 84). Intradisciplinary relationships were also seen to be affected by EMR, with nurses stating that the previously useful incidental role modeling that came from observing colleagues’ nursing documentation, patient interactions, or interprofessional collaboration were gone with EMR (Multimedia Appendix 3, quotes 85 and 86). There were also concerns voiced by nurses that graduate nursing staff or new nurses entering the profession would be limited in their critical thinking owing to their habits of following EMR prompts.

Some nurses felt that their scope of practice was limited owing to the EMR functionality limiting their actions, or the inbuilt automation that accompanies the EMR, which differed from the workflows in practice when paper-based documentation was in use. Despite this limitation, some nurses appreciated the heightened visibility of information within EMR and believed that accountability was beneficial in supporting quality patient care (Multimedia Appendix 3, quotes 87-90).

Nurses were hopeful that having the EMR throughout the health care organization would be an ongoing process of evaluation and optimization. They were grateful to be asked to participate in the research project and felt that this empowered them to provide feedback to the organization. Nurses were also asked how they could continue to develop the EMR, as they felt their voice was missing and wanted to be included in ongoing optimization and use of improvement strategies (Multimedia Appendix 3, quotes 91 and 92).

### Facilitators and Barriers to Change

#### Overview

The second stage of data analysis involved coding and mapping data to the TDF [21] (deductive analysis). A total of 1236 codes were identified and mapped to 13 of the 14 domains of the TDF [21]. Most of these data were related to barriers to nurses’ use of the EMR (819/1236, 66.26%), while just over a third were related to facilitators (417/1236, 33.74%). The underlying determinants of nurses’ behaviors were identified by mapping the TDF data to their corresponding COM-B [22] model components (capability, opportunity, and motivation). Table 1 presents exemplar quotes and the percentages of total coded barriers and facilitators for each TDF domain.

**Table 1:** Percentage of total coded barriers and facilitators for each Theoretical Domains Framework (TDF) domain with exemplar quotes (N=1236).

TDF domain	Total coded barriers (N=819), n (%)	Exemplar quotes—barriers	Total coded facilitators (N=417), n (%)	Exemplar quotes—facilitators
1. Knowledge	28 (3.4)	1. “So many mistakes being made as so many ways to interpret” [SP^a^313]	11 (2.6)	2. “If people know how to use it properly then it’s brilliant” [FG^b^8P^c^1]
2. Skills	72 (8.8)	3. “Everyone’s not proficient in EMR^d^ and different level experience” [FG6P1]	64 (15.4)	4. “We were actually talking at work um before maybe we needed just another session, like maybe 6 months into EMR, which we are doing now...which is wonderful.” [FG4P1]
3. Social or professional role and identity	82 (10)	5. “I just wonder where the, you know, the future of nursing is going to go if this is if this is how we're going to do our job. You know, we're ticking boxes.” [FG16P1]	63 (15.1)	6. “But I think most important thing would obviously still just be the patient care and then your EMR and then I think the fact that you can just double click on things means that you could spend more time with your patient and you're not stressing about documentation, cause you can always go back to it...you can just do everything you need for your patient and then document everything after.” [FG9P2]
4. Beliefs about capabilities	47 (5.7)	7. “I do default to asking the 20 year olds help me I don’t know where to find that thing. And it actually gives me a feeling of being very disempowered...I was previously a really experienced senior nurse that people would come to for help, and now I’m like, I’m useless at this.” [FG1P1]	29 (7)	8. “Where you feel you've done a good thing...where you, you find your worth in your job...if you've engaged with someone, and you've made a difference, then you go home feeling better about the job that you do.” [FG16P1]
5. Optimism	30 (3.7)	9. “The program is not user friendly.” [SP175]	52 (12.5)	10. “It’s so time efficient. It’s so easy to ah communicate with other team members through EMR um and it’s easy to look up things, everything is on the computer in front of you. So I have really, really loved using EMR.” [FG21P1]
6. Beliefs about consequences	18 (2.2)	11. “It does slow things down when it comes to the double checking and administering the medications.” [FG21P1]	0 (0)	N/A^e^
7. Reinforcement	13 (1.6)	12. “To me if the system's not as good, if not better than the system we've got, not for the coroner, but for each other, then it's not meaningful, and it's not worth it.” [FG1P1]	9 (2.2)	13. “And that was the one of the other success to be honest...policies updated.” [FG5P1]
9. Goals	4 (0.5)	14. “I actually don't know where to give advice on the EMR or where to give feedback on it.” [FG1P2]	21 (5)	15. “It’s most important to be like, very user friendly, so that we can make sure that everything's documented properly for patients and for their safety.” [FG22P1]
10. Memory, attention and decision processes	106 (12.9)	16. “Sort of find EMR more complicated than it needed to be...there's more stuff in there then you really need from a day-to-day point of view.” [FG16P1]	67 (16.1)	17. “For the most part, it’s an effective system and helps I think, the teams, nursing, allied health, everyone work a bit more cohesively because it’s all in one spot. And things can’t go missing on EMR, which, which can only benefit patient care at the end of the day.” [FG20P2]
11. Environmental context and resources	250 (30.5)	18. “I feel that my nursing assessment is less valuable, I can only record limited data that fits in to pre-determined tick boxes.” [SP231]	52 (12.5)	19. “Everything is a click away, or a couple of clicks away...information about the patient...rather than rummaging through paperwork and decipher someone's writing, whether they've been referred, whether they've been seen, everything's so yeah, clearer and easy to find” [FG20P1]
12. Social influences	27 (3.3)	20. “And I think praising that, and role modeling, that is super important. And I think that’s the risk when you've got senior nurses that are not role modeling the appropriate management or the appropriate use of the system.” [FG14P1]	44 (10.6)	21. “But then the other helpful thing is your colleagues...share with your colleagues...because they will know something that you don't know, and you know something that they don't know, and then you communicate and you, you get these things done, and it it's not as difficult.” [FG4P1]
13. Emotion	142 (17.3)	22. “I was in tears, like, three times over EMR...everyone knew I was struggling with EMR.” [FG15P1]	2 (0.5)	23. “With EMR medication mistakes have reduced to such a great extent like personally for myself, I don't have that anxiety anymore that am I looking through the chart properly and not missing anything, EMR is doing that for me.” [FG21P1]
14. Behavioral regulation	0 (0)	N/A	3 (0.7)	24. “I know some, I'm probably one of them still sort of pine a little bit for the paper but I think we, you know, things went missing with paper or things don't go missing on EMR, we have to adapt and move on, we've all sort of resigned ourselves to it.” [FG20P2]

^a^SP: survey participant number.

^b^FG: focus group interview or individual interview number.

^c^P: participant number.

^d^EMR: electronic medical record.

^e^N/A: not applicable.

#### Capability

Just over a quarter of the barriers (206/819, 25.2%) and a third of the facilitators (145/417, 34.8%) related to nurses’ use of the EMR system were associated with their capability. Barriers were often related to memory, attention, and decision processes (106/819, 12.9% of the total barriers; Table 1, quote 16). For example, cognitive overload attributed to nurses looking for information in multiple areas of the EMR was perceived to impair memory. Nurses also expressed frustration with needing to remember how to use EMR and where to find information. Capability-related facilitators involved skills such as competence and confidence in using EMR, skills development, and time and opportunities for ongoing practice, education, and training (64/417, 15.4% of total facilitators; Table 1, quote 4). Facilitators related to memory, attention, and decision processes were as follows: easier decision-making support with prompts within EMR to assist medication safety; less cognitive burden owing to improved legibility and clarity of patient information within the EMR compared with paper-based documentation; and use of and access to clinical information from anywhere within the health care organization (67/417, 16.1% of total facilitators; Table 1, quote 17).

#### Opportunity

Overall, 33.8% (277/819) of the reported barriers to and 23% (96/417) of facilitators of nurses’ use of the EMR related to opportunity. Barriers related to the environmental context and resources included nurses’ difficulty using the EMR, more time spent on the EMR than with their patients, and negative impacts of the EMR on communication (250/819, 30.5% of total barriers). The layout of the EMR was identified by nurses as problematic owing to multiple areas and ways of inputting information, and lack of standardization between organizations and between clinical areas (eg, critical care and pediatrics). EMR downtime and hardware issues, including slowness of the system, also emerged as barriers to nurses’ EMR use. Nurses felt that the EMR restricted their scope of practice; for example, selection of options rather than free-text input and restrictions related to editing or viewing of information within the system (Table 1, quote 18). Absence in the EMR of both patients’ and nurses’ narratives of care caused concerns about quality of care. Some nurses reported that the EMR contributed to negative impact on communication, with fewer clinician-clinician and clinician-patient interactions. In contrast, environmental facilitators included having a single point of access to clinical information, clinicians documenting contemporaneously, and nurses supporting their colleagues (52/417, 12.5% of total facilitators; Table 1, quote 19). Social facilitators included supportive colleagues and leadership (44/417, 10.6% of total facilitators; Table 1, quote 21).

#### Motivation

Motivation emerged as the most common behavioral driver among both barriers and facilitators (336/819, 41% of barriers and, 176/417, 42.2% of facilitators). Facilitators included the EMR supporting nurses’ professional identity and role by enabling them to do their work and prioritize patient care (63/417, 15.1% of total facilitators; Table 1, quote 6). Time to adjust to EMR and build confidence as well as previous EMR use were other facilitators of nurses’ EMR use. Support and leadership from colleagues, including senior staff and managers, were voiced as important facilitators in supporting nurses’ transition to EMR.

Nurses’ anxiety about needing to learn and use a new system, stress related to additional pressures in an already busy work environment, and fear and resistance to change with the EMR implementation emerged as emotional barriers to EMR use by nurses (142/819, 17.3% of total barriers; Table 1, quote 22). Nurses’ burnout was discussed in the context of the EMR being an additional stressor for already exhausted and stressed nurses (especially older nurses, defined as nurses >50 years of age, and those opposed to using technology).

## Discussion

### Principal Findings

The implementation and adoption of an organization-wide EMR system was an ongoing and dynamic experience for nurses who had to adapt to new ways of working. Nurses were divided in their positive and negative perceptions of the EMR and how it had impacted their work and their profession. The EMR implementation was a large organizational change that forced some nurses to reflect on their professional roles and identity and how they pictured their work moving forward.

Motivation was the underlying behavioral driver for nurses to use the EMR. Although motivation is a known element of the psychological dimension of user experience [29], many previous EMR nursing studies have simply focused on satisfaction with the system, documentation, and time spent using the EMR as influences on nurses’ EMR use [30]. Interestingly, motivation was also the main behavioral driver for nurses in pre-EMR implementation qualitative data from the same health care organization [16] and was found to be an important nurse priority for EMR implementation in a Canadian Delphi study [31].

The use of the COM-B model [22] to understand behavioral drivers for nurses’ responses to the EMR implementation identified both reflective and autonomous motivation processes that were influential and may help to explain consequences for nurses’ well-being. For example, the positive effect of motivation impacting well-being is greater when the motivation is internally driven (autonomous) rather than externally influenced [32], and autonomous motivation has been found to positively influence both well-being and behavior change in health care settings [29]. Examples of internally driven motivation in this study included nurses’ feelings of self-confidence in using the EMR and nurse-led improvements to local EMR use. Externally driven motivation was demonstrated by collegial encouragement and the completion of EMR components for fear of negative consequences. Many nurses in this study indicated low levels of autonomy and negative impact of the EMR. Possible reasons included the EMR being overprescriptive with documentation requirements and automated tasking, low visibility of some key nurses’ work, decreased time spent with patients, and not feeling involved in the EMR’s development or content. In this study, factors related to nurse well-being in relation to using EMRs were identified, as well as plausible links between nurse well-being and motivation, helping to fill a gap in the current literature [33].

The loss of nurses’ narrative owing to EMR use was raised as a concern for patient safety as well as nursing workforce retention. The absence of some clinical information and nurses’ narrative from EMR, as well as concerns about the loss of nurses’ professional identity and work visibility, created feelings of poor work satisfaction and may have had negative impacts on patient care delivery. Consistent with previous nursing literature, nurses identified time spent with patients and colleagues as well as reinforcement of their hard work and quality patient care outcomes as influential on their work satisfaction and intention to stay in their roles [34]. Unfortunately, some nurses felt their work had reduced to simply documenting for the sake of “ticking the box” on EMR, which did not fully demonstrate their work or support understanding of what was completed or still needed to be done [35]. Nurses also expressed concern about the loss of patients’ narratives in the EMR owing to changed workflows and the potential for negative impacts on patient care owing to a lack of cohesive patient information, an issue that has been previously identified in international literature [36,37].

Social influences, although viewed as an important influence on EMR use by nurses, were expressed as both positive (ie, nurses frequently providing support to each other) and negative (ie, dividing colleagues, assumptions about groups of nurses such as older nurses and younger nurses). The impact of social influences on EMR use by nurses identified in this study is consistent with findings reported in international literature and could be used as leverage by health care organizations to support nurses’ adoption and use of EMR [38,39].

End-user buy-in, through the inclusion of nurses during the design and implementation process, was intended to ensure that the system was fit for purpose [40,41]. However, at 12 to 18 months after implementation, many nurses argued that they would benefit from more time to practice and learn EMR. Although an intensive change management, training and education program was delivered to all nurses before EMR, with follow-up support after implementation, nurses identified specific scenarios and varied clinical settings as requiring further supportive measures and ongoing EMR practice. Nurses who self-identified as needing further EMR support, training, or education may benefit from engaging in organizational support available to assist with EMR knowledge or practice gaps [42]. By identifying barriers and facilitators to nurses’ EMR use, providing a safe space for nurses to voice their concerns, and feedback loops to communicate findings back to the health care organization, this study has the potential to support Australian nurses’ EMR acceptance. Supporting nurses’ use, acceptance, and knowledge of EMR may prevent EMR workarounds and deviations in workflows that can result from lack of knowledge, frustration regarding software layout, hardware slowness, and downtimes [43-45].

Nurses were concerned that it took them a long time (up until the time of data collection, ie, 12-18 months after implementation) to adjust to using the EMR in their work. The ongoing apprehensions about the EMR not meeting their expectations, inconsistent EMR use among colleagues, and cynicism regarding the legal implications of clinical documentation in the EMR indicate that these nurses may still be in the early acceptance phase. Similarly, a large Australian study examining nurses’ adoption of health care technology found that competing work demands, insufficient hardware access, and lack of support, as well as age-related decreased confidence and computer knowledge, were the main barriers to adoption [46]. Similarly, an analysis of American nurse and hospital survey data identified EMR usability and the work environment as influential factors in nurses’ adoption of EMR [47].

### Reflections on the Use of Multiple Methodological and Analyses Techniques

There were several benefits to using multiple data sources (focus group interviews, individual interviews, and free-text comments) and using complementary inductive and deductive analyses. Compared with comments made in the focus group and individual interviews where a researcher was present, many free-text comments were written using very direct and blunt language, a difference that may be owing to the anonymity provided by the survey. Differences in language between the interviews and free-text comments may also be a consequence of the Hawthorne effect. However, nurses who participated in the interviews also expressed appreciation for the opportunity to speak openly and frankly about the EMR.

The opportunities provided by multiple data sources were deemed beneficial in enabling participation and eliciting information from a wide range of participants to obtain a comprehensive understanding. Despite a limitation of survey data collection that clarification about responses could not be sought through further questioning, the free-text data provided important information about nurse workflows and aspects of nursing work not captured elsewhere. The breadth of the responses supports the transferability of the study findings to various health care settings. In addition, the use of multiple complementary data sources supported nurses’ participation and contributed honest opinions and perceptions about the effects of the EMR on them professionally and personally.

Using inductive and theory-informed deductive qualitative analyses provided a deep understanding of nurses’ perspectives and experiences and behavioral drivers, and issues influencing EMR adoption, and how it impacts their work, workflows, and the nurses personally. These results differ from previous reports in the literature that have typically examined nurses’ ease of use of EMR, satisfaction, or specific clinical outcomes related to nurses’ work and workflows with EMR systems. This study also fills a gap in the literature of theory-informed investigations of Australian nurses’ experiences of EMR.

### Limitations and Reflexivity

Limitations of this study relate to the potential for sampling bias and low response rate; a large percentage of nurses were aged >50 years, which may have influenced data interpretation. As of December 2021, only 36% of registered Australian nurses and midwives were aged >50 years [48]. Furthermore, only 22% (17/78) of the survey respondents who indicated they wished to participate in a focus group or individual interview (ie, by providing their email address at the end of the survey) could be contacted despite 2 follow-up emails. The reason for their nonresponse is not known. They may have been too busy, changed their minds, lacked interest in participating, been averse to a web-based focus group or individual interview, had privacy concerns, or been preoccupied with activities associated with the organization’s COVID-19 pandemic response. Strategies to mitigate these concerns included visiting clinical areas in person (when permitted) and explaining that nurses did not need to provide contact details to participate.

COVID-19 pandemic restrictions on research activities at the hospital sites meant that the researcher was only able to attend each hospital site in-person once. During these interactions, ward staff were often willing to briefly and honestly discuss the EMR (with the researcher), but declined to have their comments recorded or transcribed. The potential effects of the COVID-19 pandemic on nurses’ experiences of the EMR cannot be directly accounted for; however, nurses made both positive and negative comments related directly to both adapting to the EMR and its implementation in the context of the COVID-19 pandemic. The timing of COVID-19 pandemic restrictions led to an interrupted change management, training, and education program.

### Conclusions

Undertaking both inductive and deductive data analyses enabled an in-depth examination of Australian nurses’ experiences of an organization-wide full EMR system implementation. The findings revealed that barriers were most frequently related to the domains of environmental context and resources, and most facilitators were related to the domains of memory, attention, and decision processes. Motivation has emerged as the leading factor influencing nurses’ EMR adoption behaviors; hence, it should be the main component addressed in future behavior change strategies to improve EMR adoption and optimization. Implementing the EMR before the COVID-19 pandemic was seen as beneficial by some nurses owing to having a planned organizational change and facilitating access to clinical information. In contrast, it was perceived as an additional stressor by some nurses owing to isolation and use of personal protective equipment. For the benefits of EMR systems to be realized to their full potential by nurses, perceptions of the system must change from a system for retrospective documentation requiring completion compliance and taking a nurse away from providing care, to a tool that can facilitate prospective nursing decision-making that enables multidisciplinary care planning, improves clinical practice, and supports nurses’ work.

## Appendix

Multimedia Appendix 1 Semistructured interview guide.

Multimedia Appendix 2 Nurse participant demographic characteristics (N=146).

Multimedia Appendix 3 Illustrative quotes for reflexive thematic analysis themes.

## Abbreviations

COM-B: Capability, Opportunity, Motivation-Behavior
EMR: electronic medical record
TDF: Theoretical Domains Framework
