# Supplementary material for: Nurses’ Experiences After Implementation of an Organization-Wide Electronic Medical Record: Qualitative Descriptive Study
Source: JMIR Nurs. 2022 Jul 26;5(1):e39596. doi: 10.2196/39596 (PMC9328123; doi:10.2196/39596)
Supplement: Multimedia Appendix 1 [file nursing_v5i1e39596_app1.docx]

**Multimedia Appendix 1**

Semi-structured interview guide - based on the ‘4I’ model for appreciative inquiry [18]

Inquire - Key questions: Why is this important? What matters most? What works well or not so well? Purpose: to identify what features work well.

How are you feeling about the EMR?

Why is the EMR important?

What matters most?

What works well?

What doesn’t work so well?

Imagine - Key questions: How could the future be designed? What would success look like? Purpose: to challenge the status quo and identify what should be enhanced.

What would success look like?

How could the future be designed?

Innovate - Key questions: How prepared or ready are we for the ideal design? What needs to change to become a reality? What factors may effect these required changes? Purpose: to identify what changes are required to enable the ideal to occur.

Did you feel prepared?

Did you feel ready?

What needs to change?

Implement - Key questions: What is required to implement these innovative ideas or designs? Who needs to be involved to implement these innovative designs? What factors may affect the implementation of these innovative designs? Purpose: identify how to implement innovation into reality.

Can you identify any facilitators to using the EMR?

Can you identify any barriers to using the EMR?
