# Supplementary material for: Nurses’ Experiences After Implementation of an Organization-Wide Electronic Medical Record: Qualitative Descriptive Study
Source: JMIR Nurs. 2022 Jul 26;5(1):e39596. doi: 10.2196/39596 (PMC9328123; doi:10.2196/39596)
Supplement: Multimedia Appendix 2 [file nursing_v5i1e39596_app2.docx]

**Multimedia Appendix 2**

**Table 1.** Nurse participant demographic characteristics (N =146).

| Characteristic | | Post-EMR^a^ implementation nurse participants, n (%) | Median (IQR) | Range |
| --- | --- | --- | --- | --- |
| **Age (years)** | | | 44 (32-56) | 22-70 |
|  | 20-29 | 28 (19.2) |  |  |
|  | 30-39 | 30 (20.5) |  |  |
|  | 40-49 | 29 (19.9) |  |  |
|  | 50-59 | 40 (27.4) |  |  |
|  | 60-69 | 15 (10.3) |  |  |
|  | 70-79 | 2 (1.4) |  |  |
|  | Missing | 2 (1.4) |  |  |
| **Gender** | | | N/A^b^ | N/A |
|  | Man | 12 (8.2) |  |  |
|  | Woman | 132 (90.4) |  |  |
|  | Other or prefer not to say | 1 (0.7) |  |  |
|  | Missing | 1 (0.7) |  |  |
| **Nurse classification** | | | N/A | N/A |
|  | Registered nurse (graduate) | 12 (8.2) |  |  |
|  | Registered nurse (grade 2) | 45 (30.8) |  |  |
|  | Enrolled nurse | 12 (8.2) |  |  |
|  | Clinical nurse specialist | 42 (28.8) |  |  |
|  | Associate nurse manager | 24 (16.4) |  |  |
|  | Nurse manager | 7 (4.8) |  |  |
|  | Educator | 4 (2.7) |  |  |
| **Years worked as a nurse** | | | 20 (7-32) | 0-52 |
|  | 0-4 | 22 (15.1) |  |  |
|  | 4.5-9 | 24 (16.4) |  |  |
|  | 10-14 | 16 (11.0) |  |  |
|  | 15-19 | 8 (5.5) |  |  |
|  | 20-24 | 19 (13.0) |  |  |
|  | 25-29 | 7 (4.8) |  |  |
|  | 30-34 | 15 (10.3) |  |  |
|  | 35-39 | 14 (9.6) |  |  |
|  | 40-44 | 12 (8.2) |  |  |
|  | 45-49 | 2 (1.4) |  |  |
|  | 50-54 | 3 (2.1) |  |  |
|  | Missing | 4 (2.7) |  |  |
| **Highest level of education, n (%)** | | | N/A | N/A |
|  | High school | 1 (0.7) |  |  |
|  | Certificate or diploma | 24 (16.4) |  |  |
|  | Degree | 54 (37.0) |  |  |
|  | Postgraduate certificate/diploma | 55 (37.7) |  |  |
|  | Higher degree (master’s or PhD) | 9 (6.2) |  |  |
|  | Missing | 3 (2.1) |  |  |
| **Hours worked (average per fortnight)** | | | 64 (48-70) | 48-70 |
|  | 17-32 | 21 (14.4) |  |  |
|  | 33-48 | 25 (17.1) |  |  |
|  | 49-64 | 60 (41.1) |  |  |
|  | 65-80 | 32 (21.9) |  |  |
|  | >80 | 5 (3.4) |  |  |
|  | Missing | 3 (2.1) |  |  |
| **Work area** | | | N/A | N/A |
|  | Medical / surgical ward | 27 (18.5) |  |  |
|  | Critical care | 74 (50.7) |  |  |
|  | Pediatrics | 7 (4.8) |  |  |
|  | Subacute | 22 (15.1) |  |  |
|  | Procedural units | 13 (8.9) |  |  |
|  | Missing | 3 (2.1) |  |  |
| **Site** | | | N/A | N/A |
|  | A | 17 (11.6) |  |  |
|  | B | 18 (12.3) |  |  |
|  | C | 18 (12.3) |  |  |
|  | D | 55 (37.7) |  |  |
|  | E | 9 (6.2) |  |  |
|  | F | 25 (17.1) |  |  |
|  | Missing | 4 (2.7) |  |  |

^a^EMR: electronic medical record.

^b^N/A: not applicable
