# Supplementary material for: Nurses’ Experiences After Implementation of an Organization-Wide Electronic Medical Record: Qualitative Descriptive Study
Source: JMIR Nurs. 2022 Jul 26;5(1):e39596. doi: 10.2196/39596 (PMC9328123; doi:10.2196/39596)
Supplement: Multimedia Appendix 3 [file nursing_v5i1e39596_app3.docx]

**Multimedia Appendix 3**

**Table 2.** Illustrative quotes for reflexive thematic analysis themes.

| Theme, subtheme, and context | | | Exemplar quotes |
| --- | --- | --- | --- |
| **1. An unintentional divide** | | | |
|  | **1. Then and now** | | |
|  |  | **Nurses’ expectations about the EMR^a^ assisting their work not met** | |
|  |  |  | 1. “Overwhelmed and disappointed” [FG2P1^b,c^] |
|  |  |  | 2. “I had such high hopes for EMR” [SP137^d^] |
|  |  | **EMR implementation was stressful** | |
|  |  |  | 3. “Exciting and exhausting” [SP364] |
|  |  |  | 4. “EMR was very challenging at first” [SP159] |
|  |  | **Mixed feelings about the timing of the implementation (all components of documentation changed to electronic)** | |
|  |  |  | 5. “Really good (health care organization) went fully electronic in one go” [FG5P1] |
|  |  | **Nurses’ attitudes and culture** | |
|  |  |  | 6. “The implementation of the EMR was so dependent on the culture of the ward or the area or at the time, that if the ward had a good culture, they just managed to pick it up, they all helped each other.” [FG1P2] |
|  |  | **Nurses with confidence using technology were better at adopting and accepting EMR** | |
|  |  |  | 7. “Everyone is different with computers.” [FG12P1] |
|  |  |  | 8. “I do not think that you can just fluff your way through it... actually invest the time and do the learning.” [FG3P1] |
|  |  | **Took time for EMR adoption and acceptance** | |
|  |  |  | 9. “I feel good using the EMR and getting better each day.” [SP332] |
|  |  |  | 10. “Very stressful in the start but now I really like using EMR.” [SP315] |
|  |  |  | 11. “I do feel like as time goes on, I am getting better... exposure and use.” [FG6P2] |
|  |  | **Not using EMR to full capabilities (eg, nurses able to customize EMR planner)** | |
|  |  |  | 12. “I think it’s become a focus, rather than a tool that we are supposed to be using to make our life easier, to make our job easier, to make our interactions with our patients easier.” [FG16P1] |
|  |  | **Inadequate training** | |
|  |  |  | 13. “No, I was not prepared... one day of training.” [FG4P1] |
|  |  | **Grateful for ongoing support measures from the organization (web-based materials, in-person support, phone support)** | |
|  |  |  | 14. “There’s a lot of stuff on online education portal), so I should probably do some of them again, like a refresher… ongoing education would be good” [FG7P1] |
|  |  |  | 15. “If we did not have... super users and champions on the wards... it would have been a lot of struggle.” [FG5P1] |
|  |  |  | 16. “Sometimes I do get extremely frustrated... teaching other people... I love it... but when you have your own sick patient... it’s just sometimes a bit overwhelming.” [FG6P1] |
|  |  | **Difficult for some nurses to adapt to EMR (less time spent using the system or less time with in-person support)** | |
|  |  |  | 17. “The people who work (part-time)... they really struggle.” [FG1P1] |
|  |  |  | 18. “Super users, your technician team was already there always 24/7... a lot of support was there during the changes.” [FG5P1] |
|  |  | **Suggestions for improvements to support nurses’ work and workflows** | |
|  |  |  | 19. “It will be helpful if there’s an alert like when they change the medication, you can see that.” [FG10P4] |
|  | **2. Clicking or caring** | | |
|  |  | **Divide in nurses’ priorities—the EMR or their patient** | |
|  |  |  | 20. “People are so obsessed with clicking and ticking down tasks so they've forgotten to look at the patient or look after them.” [FG8P1] |
|  |  |  | 21. “You're nursing the WOWs these days, you're not nursing the patients.” [FG15P1] |
|  |  | **Completing EMR documentation for fear of negative response** | |
|  |  |  | 22. “I am concerned that people are more concerned about ticking the boxes and not doing the doing.” [FG11P1] |
|  |  |  | 23. “Cause they want to get rid of that red tile... everyone's trying to do their best to get rid of that red tile.” [FG19P1] |
|  |  | **Strong positive or negative feelings toward using the EMR related to device integration, communication, and medication safety** | |
|  |  |  | 24. “There's good and bad things about EMR.” [FG1P2] |
|  |  |  | 25. “Whole point of EMR was to have data integration but we don't so it's just doubled our workload.” [FG8P1] |
|  |  |  | 26. “Easy to communicate with the multidisciplinary team.” [SP213] |
|  |  |  | 27. “Communication has deteriorated since its introduction.” [SP75] |
|  |  |  | 28. “EMR is good with for specifically medication administration and picking up medication errors.” [SP117] |
|  |  |  | 29. “The meds chart is awful to read and I am constantly worried I have forgotten something or will make an error.” [SP32] |
|  |  |  | 30. “I quickly can see what's ordered... everything in EMR.” [FG5P1] |
|  |  | **Emotional responses to positive work environment** | |
|  |  |  | 31. “Our managers... very much on board with EMR, they'd been consulted, as we understand it into like the creation of it, so they really championed it, and I think we had a really positive outlook around it.” [FG20P2] |
|  |  | **Positive EMR experiences related to supporting nurse documentation, less duplication, improved legibility, accessibility, and removing paper** | |
|  |  |  | 32. “The ability to do your own research on the patient... look back through the documentation.” [FG1P1] |
|  |  |  | 33. “I think it's such a convenient and you know, time saving documentation um with your patient care.” [FG6P1] |
|  |  | **Negative feelings related to poor experiences** | |
|  |  |  | 34. “Not user-friendly” [SP137] |
|  |  |  | 35. “Families try and come sneak, look at it... have to be weary when they're around.” [FG10P2] |
|  |  |  | 36. “Information overload as well as confusion.” [SP111] |
|  |  | **Workarounds owing to constraints of EMR documentation or time to complete EMR documentation** | |
|  |  |  | 37. “Always looking for the right place to document something.” [SP30] |
|  |  |  | 38. “Documentation... a lot of the time like I need to pick what ‘best fits’ rather than... clinical situation.” [SP321] |
|  |  | **Nursing time spent correcting EMR** | |
|  |  |  | 39. “Very easy to miss or forget documenting something as a nurse as you have to put the same information in multiple different places.” [SP175] |
|  |  |  | 40. “90% time spent correcting orders” [FG2P1] |
|  |  | **EMR negatively impacts nurse autonomy** | |
|  |  |  | 41. “Takes away nuance, personal judgement and critical thinking” [SP330] |
|  |  |  | 42. “EMR doesn’t value... the knowledge of the nursing staff” [FG1P2] |
|  |  | **Nursing documentation not valued or of quality** | |
|  |  |  | 43. “Clickboxing... doesn't make documenting meaningful” [FG1P1] |
|  |  |  | 44. “Quality of meaningful documentation has decreased” [SP32] |
|  |  | **EMR is a barrier between the nurse and the patient** | |
|  |  |  | 45. “Barrier between the nurse and the patient” [FG12P1] |
|  |  |  | 46. “EMR is really sort of a bit of a barrier to me feeling like I've properly looked after the patient, or... properly documented everything that I've done.” [FG19P1] |
|  |  | **EMR hardware and software disrupted nursing care** | |
|  |  |  | 47. “Most shifts, I would easily waste 45 minutes dealing with a computer.” [FG15P1] |
|  |  |  | 48. “EMR downtime... it's quite stressful.” [FG6P1] |
|  |  | **What matters most to nurses** | |
|  |  |  | 49. “The most important thing is always going to be patient care to us and EMR helps facilitate that” [FG20P2] |
|  |  |  | 50. “Giving the proper care, having the time to give the care” [FG11P1] |
|  | **3. Consequences and assumptions** | | |
|  |  | **Negative impact on nurse well-being, work satisfaction and retention** | |
|  |  |  | 51. “We've had two nurses leave over EMR.” [FG18P1] |
|  |  |  | 52. “Seriously impacted my job confidence, and enjoyment, and I am looking at leaving the acute area because of it.” [SP343] |
|  |  |  | 53. “I was part of the super user... I lost sleep over how difficult it was to implement the EMR into our daily work lives.” [SP12] |
|  |  | **Other stressors affecting nurses including the COVID-19 pandemic** | |
|  |  |  | 54. “I feel there are other factors that contribute more to the feeling of being burnt out, than using EMR.” [SP350] |
|  |  |  | 55. “COVID-19 has also played a huge role in this as well.” [SP215] |
|  |  | EMR useful during the COVID-19 pandemic | 56. “Good to have during pandemic.” [SP81] |
|  |  | **EMR knowledge or use dividing nurses** | |
|  |  |  | 57. “The great divider on the ward, it is the, the new ‘bully’ of the workplace” [SP166] |
|  |  |  | 58. “People who don't know how to use it... will be documenting incorrect things so that causes problems.” [FG21P1] |
|  |  |  | 59. “I do default to asking the 20-year-olds help me... gives me a feeling of being very disempowered... I was previously a really experienced senior nurse that people would come to for help, and now I'm like, I'm useless at this.” [FG1P1] |
|  |  | Assumptions about younger and junior staff | 60. “Junior staff who okay are probably more tech savvy than I am” [FG1P1] |
|  |  | **Older nurses more negatively impacted by EMR** | |
|  |  |  | 61. “For the older staff... a little bit more TLC would be worthwhile... it is harder for them.” [FG14P1] |
|  |  |  | 62. “Those middle-aged people who take a bit longer to understand.” [FG5P1] |
|  |  | Surprise at older nurses’ adapting to EMR | 63. “Surprisingly, so many of the older nurses are actually really quite adjusting.” [FG14P1] |
| **2. This time, it’s personal** | | | |
|  | **1. A constantly changing profession** | | |
|  |  | **EMR was a huge change for nurses** | |
|  |  |  | 64. “Everybody has that, you know, resistance, fear of change because it challenges you know, their feelings of self-worth.” [FG14P1] |
|  |  |  | 65. “The reality is... it’s a profession that’s constantly changing. And if you do not change with it, then you will get left behind.” [FG16P1] |
|  |  | **Different responses and resistance to change** | |
|  |  |  | 66. “Didn’t feel confident... there was a lot of resistance to let go of the paperwork.” [FG20P1] |
|  |  |  | 67. “Getting rid of paper handover was an unnecessary evil.” [SP355] |
|  |  | **Positive change from paper to electronic** | |
|  |  |  | 68. “Could never go back to paper” [FG9P1] |
|  |  |  | 69. “So much could get lost in those old notes... errors from not being able to read clearly... harm to patients if things weren't communicated well, or clearly... it was definitely time to move forward.” [FG14P1] |
|  |  | **Time taken to document on EMR** | |
|  |  |  | 70. “On paper, we were much quicker” [FG19P1] |
|  |  |  | 71. “Old charts... everything was in front of you instead of having to flick to different screens.” [SP117] |
|  |  | **Comparing EMR systems** | |
|  |  |  | 72. “Their EMR seems to be a little bit less clunky, less... things to choose.” [FG19P1] |
|  |  |  | 73. “Less tabs... it's grouped more logically... more user friendly in that aspect... less clicky... the medication colors are a little bit different... easier to read.” [FG6P1] |
|  |  | **Fear of negative consequences related to legality and visibility of documentation** | |
|  |  |  | 74. “I feel that I would be unable to defend myself in a court of law with EMR” [SP75] |
|  |  |  | 75. “Harder protecting ourselves or our patients” [FG2P1] |
|  |  |  | 76. “And I can't believe more people have not made mistakes... I'm constantly terrified.” [FG1P1] |
|  |  | **Adapting to a different system** | |
|  |  |  | 77. “Poorly laid out” [SP299] |
|  |  |  | 78. “EMR would have been more helpful to me IF - The organization had bought an appropriate package that is not American with American terminology” [SP75] |
|  |  | **Consultation in EMR development and implementation** | |
|  |  |  | 79. “Did not seem that there was much nursing input in creating the program” [SP159] |
|  |  |  | 80. “EMR has not been designed with the user in mind or in consultation with the user groups, it is clunky and difficult to use” [SP327] |
|  | **2. What will nursing become** | | |
|  |  | **Nursing has become depersonalized** | |
|  |  |  | 81. “Lose that ability to get a feel for how that nurse cared for that person.” [FG11P1] |
|  |  |  | 82. “EMR... has made nursing more difficult and very task orientated instead of it being about the patient it’s about the task.” [SP342] |
|  |  | **Differences in documentation between disciplines** | |
|  |  |  | 83. “We're both working together to treat the patient but we're not kind of looking at the same documentation.” [FG7P1] |
|  |  |  | 84. “Now a year in... feedback from medical team is saying, look, I can't figure out why this was done and what actually happened, I wish there was a nursing note.” [FG20P1] |
|  |  | **Loss of role modeling** | |
|  |  |  | 85. “Junior nurses or your younger nurses, are not having that opportunity to role model against... a more senior nurse... because they've got to get back to ticking a box... back onto a computer.” [FG16P1] |
|  |  |  | 86. “Staff are losing their ability to critically think, assess their patients, they are waiting for the EMR to tell them what to do.” [SP121] |
|  |  | **Nurses’ scope of practice limited** | |
|  |  |  | 87. “EMR can be too restrictive with certain tasks and we are unable to make changes” [SP334] |
|  |  |  | 88. “Some areas of the EMR where I feel we should be given more autonomy... prior to the EMR it was a nursing decision” [SP285] |
|  |  | **Accountability of documentation or actions within EMR** | |
|  |  |  | 89. “With EMR is it really does make people accountable for their actions and their times” [FG14P1] |
|  |  |  | 90. “It's very easily traceable who did it, what time” [FG20P1] |
|  |  | **Ongoing process of evaluation and optimization** | |
|  |  |  | 91. “You're taking feedback from us and things will get even better” [FG21P1] |
|  |  |  | 92. “I am happy to, you know, give some ideas... how they could make it a little bit easier” [FG19P1] |

^a^EMR: electronic medical record.

^b^FG: focus group interview or individual interview number.

^c^P: participant number.

^d^SP: survey participant number.
